# Simulation in thoracic surgery—A mini review of a vital educational tool to maximize peri-operative care and minimize complications

**DOI:** 10.3389/fsurg.2023.1146716

**Published:** 2023-05-03

**Authors:** Hasanali David Walji, Steven Aaron Ellis, Antonio Eduardo Martin-Ucar, Luis Hernandez

**Affiliations:** ^1^ Department of Cardio-Thoracic Surgery, University Hospitals Coventry and Warwickshire, Coventry, United Kingdom; ^2^ Department of Medical Education, University Hospitals Coventry and Warwickshire, Coventry, United Kingdom

**Keywords:** peri-operative complications, thoracic surgery, medical simulation, medical education, post operative complication, surgical simulation and training

## Abstract

Thoracic surgery is an increasingly expanding field, and the addition of national screening programs has resulted in increasing operative numbers and complexity. Thoracic surgery overall has an approximately 2% mortality and 20% morbidity with common specific complications including persistent air leak, pneumothorax, and fistulas. The nature of the surgery results in complications being unique to thoracic surgery and often very junior members of the surgical team feel underprepared to deal with these complications after very little exposure during their medical school and general surgical rotations. Throughout medicine, simulation is being increasingly used as a method to teach the management of complicated, rare, or significant risk occurrences and has shown significant benefits in learner confidence and outcomes. In this mini review we explain the learning theory and benefits of simulation learning. We also discuss the current state of simulation in thoracic surgery and its potential future in aiding complication management and patient safety.

## Introduction

The origins of thoracic surgery date back at least to 1,499 A.D. in Bologna when a chirurgeon called Rolandus performed a wedge section of infected lung tissue (1). Ironically Rolandus, a Christian man, would likely have refuted this suggesting thoracic surgery dates to the dawn of man when Adam's rib was resected to create Eve (2). Despite the disputed age of the specialty, thoracic surgery is at present undergoing a noteworthy rebirth throughout the United Kingdom (3).

The significant increase in workload has arisen from the prominence of worldwide lung cancer screening programs which have shown to reduce overall lung cancer mortality (4). Furthermore, the research into rib fixation (5, 6), vaping related lung pathologies (7, 8), increase in metastases resection (9) and the unknown entity of COVID-19 related lung injury are all bringing potential volumes of operative work. This surge in work will require a larger and more highly trained thoracic workforce than ever before.

Patients undergoing cardio-thoracic surgery suffer a higher mortality when compared to all other surgical specialties (10, 11). Overall, thoracic surgery has an approximately 2% mortality and 20% morbidity. It has also been reported that death in all non-cardiac surgery patients is most likely to occur post-operative period, rather than in the intra-operative phase (12). In view of this, further training in post-operative management could improve morbidity and mortality in patients.

When considering the post-operative period, patients who have undergone thoracic surgery are unique as if they develop an acute illness, their management is often different to that of any of their surgical or general medical patient counterpart. An example of this is if a patient suffers with post-operative atrial fibrillation, the current UK NICEs guideline (NG196) uniquely has a specific section for the atrial fibrillation management post cardio-thoracic surgery (13). Furthermore, specific thoracic surgery patients, such as double lung transplants via sternotomy, have an entirely separate cardiac arrest management protocol compared to all other non-cardiothoracic surgery patients as the usual Advance Life Support algorithm is not suitable (14). Although, the previously mentioned post operative complications are not the most common, they are arguably some of the most significant in terms of potential patient harm.

In view of the above position, it is imperative to ensure the medical staff attending these patients are well trained in management of these unique emergencies. A potential method of training staff is in the simulation learning environment.

## What is simulation?

Simulation is defined as:

*“A technique that creates a situation or environment to allow persons to experience a representation of a real event for the purpose of practice, learning, evaluation, testing, or to gain understanding of systems or human actions”* (15).

Simulation has it's reported use since at least 1,560 A.D. when the miliary writings of the German aristocrat, Reinhard Graf Zu Solms, were published posthumously. The 7th volume of “Kriegsbeschreibung” was dedicated to a card-based war game to simulate battlefield decisions for commanding officers (16). It would be almost 400 years later when the European toymaker, Åsmund S. Lærdal, presented the first widespread medical simulation tool in the Resusci Anne. This was a mannequin designed to be used in cardiac arrest simulations, and to this day is used throughout the world in providing lifesaving training (17).

Over the last 60 years, simulation has taken a leading role in medical education. It is now incorporated into the curriculum for medical students, foundation doctors, and subspecialty trainees across disciplines as set out in the General Medical Councils “*Promoting Excellence: Standards for medical education and training*” (18). It is used to teach a variety of technical and non-technical skills via a range of methods and fidelities (19).

Fidelity is defined as the degree to which a simulated activity reflects real life (20). In surgery, common low-fidelity simulations include the use of suturing pads and laparoscopic box simulators; whereas high-fidelity simulation occur in the form of cadaveric and live animal models. There is an ongoing debate around the merits of low verses high fidelity simulation although current literatures suggests neither shows superiority (21–23).

Some educationalists argue the fidelity of a simulation is inconsequential as it is the individuals experience which confers the merit of the learning activity. This notion forms the basis of the educational theory as to why simulation is valuable, experiential learning.

## Simulation learning theory and importance of debrief

**Figure 1:**
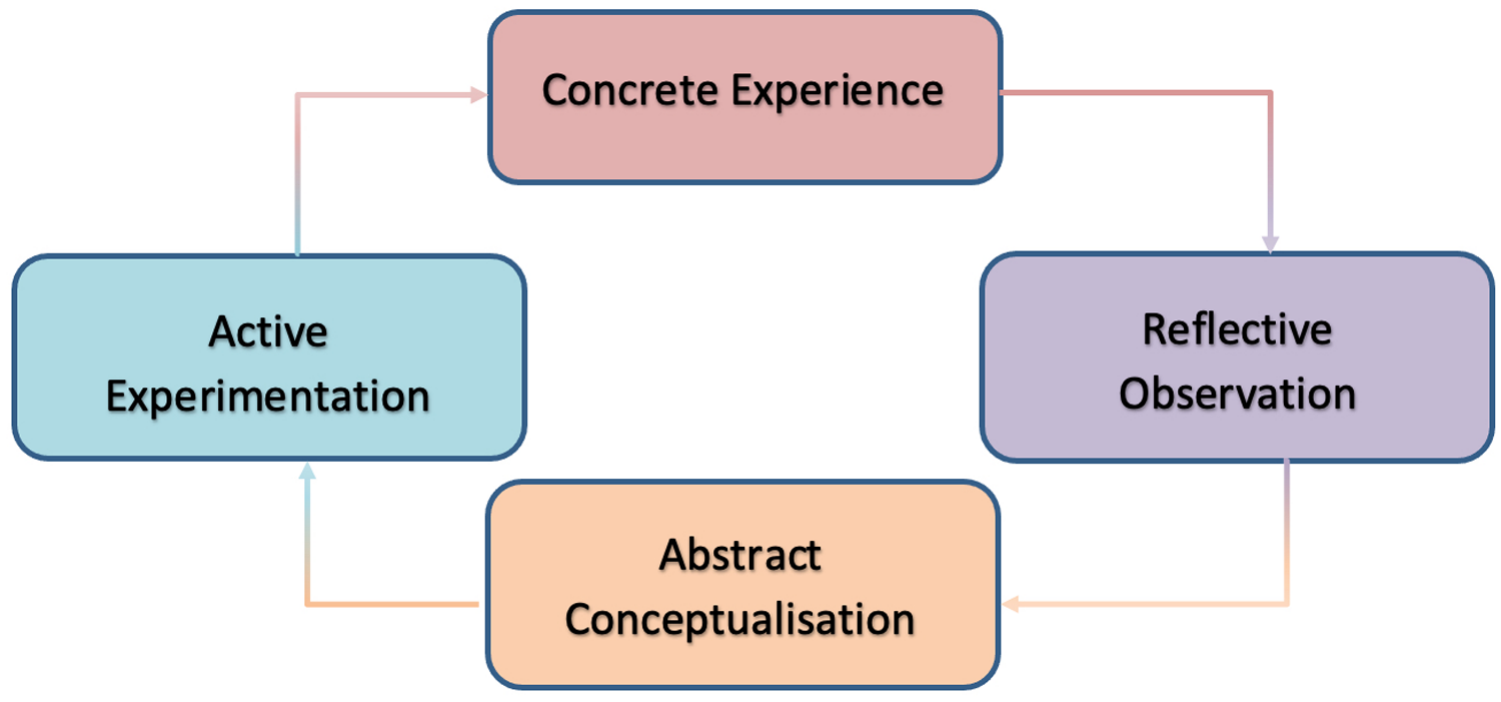
Kolb's learning cycle (24).

The Experiential Learning Theory (ELT) is the theoretical framework that forms the basis of simulation learning. Kolb (24) explains experiential learning theory exists arounds the central notion that one learns by interaction with a reality rather than reading or listening about it; essentially one learns through experience. Kolb's learning cycle is a four-stage cycle that highlights how one learns *via* experience (Figure 1).

Experiential learning theory and Kolb's learning cycle are useful concepts when revieing simulation as the basis of simulation is to create an environment for the learner to interact with as a core part of their learning (25). The simulation forms the concrete experience stage of the cycle.

Kolb (24) goes on to suggest that experience alone is not enough to learn, but one must reflect on that experience in order to ensure the learning points are free from subjectivity or bias. In the context of simulation, this is where debrief is used. Debrief forms the reflective observation and abstract conceptualization stages of the cycle.

Fanning and Gaba (26) define debrief as “facilitated or guided reflection in the cycle of experiential learning”. Several approaches to debrief exist which can be categorized by the timing (i.e., during or after); method of delivery (i.e., conversations or written) and by the guide (i.e., facilitator or learner) (27). Currently, there is very little robust evidence to suggest which method is the best with Sawyer et al. (27) arguing the process of debrief is more important than the method. Adding to this, Shinnick (28) demonstrates that there is a significant improvement in learner-centered outcomes if debrief occurs. Furthermore, Crookall (29) and Stewart (30) argue that it is unethical to provide simulation without debrief as the learner needs an opportunity to discuss their experience.

Irrespective of the method of chosen, it is widely accepted the role of the facilitator is key to the debrief process and as such, it is important to select the most appropriate person to lead said debrief (26). In a study conducted by NASA (31), learners' perception of the facilitators debriefing skills was the highest correlating factor to the overall perceived quality and credibility of the simulation. Similarly, a study in nursing students comparing subject matter experts to non-subject matter experts in debriefing showed no benefit to the perception of the simulation's effectiveness (32). These studies suggest the facilitators debriefing skills are more important than the level of expertise the facilitator has on the subject matter. This is likely because facilitators in debrief place themselves as equals in the process, guiding learning through points, rather than authorities on the subject matter.

Conflictingly, some suggest a subject matter expert is important in debriefing simulations surrounding specialties areas (such as thoracic surgery) as it provides grounded knowledge and credibility to the process (31, 33, 34). Though there is no consensus in the literature, it is conceivable the correct choice of debrief facilitator is situation dependent. In simulations whereby learning points are more based around non-technical skills, it is likely the subject matter expertise of the facilitator is far less important compared to ones which are focused on technical skill learning.

When specifically reviewing the literature surrounding surgical simulation, the term facilitator is often replaced with mentor. Many studies determine a mentor as a senior member of the surgical team, or a subject matter expert, who can provide expert guidance in a specific simulation. The Standing Conference on Postgraduate Medical and Dental Education (SCOPME) (35) defines mentoring as:

“*The process whereby an experienced, highly regarded, empathic person (the mentor), guides another individual (the mentee) in the development and re-examination of their own ideas, learning, and personal and professional development. The mentor who often, but not necessarily, works in the same organisation or field as the mentee, achieves this by listening and talking in confidence to the mentee”*

This above definition suggests the use of the term mentor / mentorship in many surgical simulations paper is an oversight as mentorship is a process rather than a single event. Currently, there is very little published in the literature regarding the use of long-term mentorship in surgical simulation, though mentorship in actual surgery is well established (36, 37). The use of mentors to provide consistent debrief to the same learners or groups of learners in both a simulated environment and subsequently real theatre environment is a topic which needs to be further explored.

## Benefits of simulation education

The use of simulation in surgical education has a number of benefits. These include the ability to repeatedly practice clinical scenarios without risk to patients and create rare and complex scenarios in a controlled environment with the benefit of debrief. The consistent experience which simulation provides, also allows for equability learning opportunities across varying learner groups (38).

A number of studies exist showing the use of simulation based education resulting in improved learning and outcomes (39). Many of these studies directly compare conventional clinical teaching against simulation for development of technical and non-technical skills. A study by Langhan et al. (40) showed improved outcomes in resuscitation comparing the use of simulation against conventional education techniques, this effect was noted to be persistent after 3 months. Similarly, two studies by Wayne et al. (41, 42) showed students who underwent simulation learning compared to conventional teaching methods had improved cardiac arrest management.

The use of simulation in education is not without its drawbacks. Many of these concerns are logistical in terms of cost, infrastructure and time (43). Learner specific concerns such as participants acting in a more caviler manner due to their awareness of a lack of specific consequence to their actions in simulation (44). Finally, there is a concern regarding overconfidence. In the 18 months preceding this mini-review alone, multiple studies have been published which show simulation based education significantly improves learners confidence (45–50). Interestingly, there is very little published as to whether improved learner confidence can translate to overconfidence. Overconfidence is a form of cognitive bias and has been shown to create a blind spot in decision making. One study has demonstrated overconfidence as a negative outcome after simulation (51). This blind spot for healthcare practitioners has been linked to overly risk-taking behavior, inappropriate clinical decision making and worse patient outcomes (52–55).

Further research needs to be conducted into the prevalence of overconfidence after simulation as this could be creating a pervasive cognitive bias linked to suboptimal clinical care.

## Current state of simulation in thoracic surgery

In 2007, the Visioning Simulation Conference (VSC) was held under the sponsorship of multiple representative organizations throughout thoracic surgery including the European Association for Cardiothoracic Surgery (EACTS), American Association For Thoracic Surgery (AATS) and Society Of Thoracic Surgery (STS) (56). This conference aimed to assess the educational needs of the specialty and how simulation could be used to meet those needs. It established how simulation can be used to minimize patient harm and improve outcomes. The conference highlighted the need for simulation in three broad areas:

1. Technical skills
2. Non-technical skills
3. Emerging technologies.

Since the conference, there has been increasing body of published works in the implementation of simulation in cardiothoracic surgery; though the number of thoracic surgery simulations is overshadowed by the volume of simulations developed by our cardiac surgery counterparts. Simulation has been successfully implemented into the curriculum for Cardiothoracic Surgery trainees in the United Kingdom. This comprise of a series of centrally run sessions throughout training, however these are generally only run annually (57). Other thoracic and cardiac surgery organizations have also successfully added simulation to their curricula, however these again are often limited to singular “Bootcamps” sessions rather than as part of continuous educational activity (58–60).

Importantly, organizations which have instituted simulation as part of their curriculum, have both specific technical and non-technical learning events/outcomes.

### Technical skills simulation

Technical skill simulation is the most common form of thoracic surgery simulation cited in the literature. It has been used to teach a range of surgical specific skills across training levels and locations.

Historically, human cadaveric issue has been favored to teach anatomy and surgical dissection throughout medical education. Cadavers have the benefit of providing an anatomically correct and a realistic tissue experience for the learner. Cadaveric tissue will, however not respond in the same way as living issue. In thoracic surgery, attempts have been made to improve this experience through careful cadaver preparation via synthetic gels insertion with good result (61). However, due to the high cost, ethical concerns, and reduced availability, cadaver use has been widely replaced with animal models. The use of animal tissue is possible in technical simulation due to the similarities of porcine lung anatomy and human (62).

Tedde et al. (63) describe a technical simulation course using anaesthetized swine. It provided for a realistic surgical experience and allowed for the learners to development an understanding of the “technical peculiarities” observed during video assisted thoracoscopic (VATs) lobectomy. Though this method is one of the highest fidelity methods seen in the literature, is it not widely practiced due to the high cost and facility requirement to run a simulation of this nature.

Jimenez and Gomez-Hernandez (64) describe a more common style of cadaver animal simulation. They demonstrated the use of commercially available porcine heart-lung blocks as viable models in teaching VATs lobectomies in a controlled environment. They describe their departmental policy requiring 25 simulated lobectomies to be performed prior to a trainee starting to operate on a patient. This allows for a reduced learning curve when operating on patients and as such reduces potential patient harm. Similar porcine simulations have also been used in training Mexican thoracic surgery residents, with high levels of satisfaction reported both by trainee and trainer (65).

Animal models also allow for rare and immediate life-threatening pathologies to be practiced when training of this nature is difficult to provide in the clinical environment safely and consistently. Du et al. (66) demonstrated the use of porcine models to teach emergency management of penetrating thoracic injuries. Outside of simulation this would be difficult to teach consistently. They established that animal models can be made with an acceptably low coefficient of variation, provide a post-training performance improvement, and have a broad use of thoracic surgery training applications.

Synthetic material can be criticized due to its low fidelity. Despite this, Synthetic tissue simulations have been used to teach a variety of procedures and have established benefits. Complex printed synthetic biostructures have been used to teach and evaluate surgical ability to perform VATs lobectomy across surgeons of varying experience (67). Furthermore, synthetics materials have been repeatedly used to teach multiple learners emergency thoracic skills; such as chest drain insertion and thoracocentesis (68). Finally, synthetics, low-fidelity, low-cost simulation has been used to set up thoracic surgery training in resource poor environments (69, 70).

The use of three-dimensional virtual reality to teach technical skills is a rapidly developing area in surgical simulation. Virtual reality based simulation have the potential to provide infinite reproducibility, with the possibility of training without the need for a facilitator as the software can provide said supervision (71). Currently, there is limited data on its use in thoracic surgery. Small studies have shown to confer a benefit in technical skill acquisition, student satisfaction and competency assessment (72–75).

### Non-technical simulation

Non-technical skills are group of wider skills required by surgeons in order to provide effective patient care. They can be divided into several categories such as social, cognitive, and personal skills (76). Failures in effective communication, teamworking, leadership, decision-making, situational awareness and coping with stress are all examples of failures of non-technical skills. They have been attributed as one of the main causes of error in the operating department, with some studies showing technical errors contribute to less than 5% of all operating department errors (77).

Bierer et al. (78) have reported the development of an *in situ* thoracic surgery crisis simulation aimed at developing non-technical skills. They created an emergency post-operative airway obstruction, with the case testing communication and decision-making skills alongside other technical and non-technical skills. Using validated scoring systems (NOTSS and STEPPS2) they have demonstrated improved learner-centered outcomes.

In 2013, Burkhart et al. (79), created a program to teach the use post-cardiotomy extracorpeal membrane oxygenation (ECMO). From the outset of their program, they highlighted the importance of both the technical and non-technical aspects of running an ECMO service and as such created a non-technical simulation to reflect this. The post-course review suggests learners had developed both a better technical understanding of ECMO and the behaviors required to manage complications. This program highlighted the ability to introduce both technical and non-technical learning when introducing new surgical concepts.

Tsitsias et al. (80) also created a similar program to teach airway emergencies, however this was a combined program for both anaesthetic and thoracic surgery trainees. Though they did not report any validated outcome measures, they did report high levels of satisfaction from participants suggesting an multi-disciplinary team (MDT) based learning experience can be effective.

### Emerging technologies (robotic surgery)

The final area of simulation learning suggested by the VSC was in emerging technologies.

In 2002 Melfi et al. (81) presented their case series of lung resection via robotic assisted thoracic surgery (RATs). Since its inception, RATs has increasingly been chosen as the method for lung resection, with up to 20% of lobectomies in the United States conducted via RATs (82). Furthermore, the prevalence of RATs has increased year-on-year throughout the world and subsequent new robots and methods (such as Uniportal-RATs) have been described (83, 84).

As a new technology in the era of simulation, several simulations based around the use of RATs have been described. Whittaker et al. (85) have described the development and validation of a virtual reality simulator for robotic training. They noted the very nature of robotic surgery lends itself to simulated training and as such was received well by participants.

As the lead surgeon is not scrubbed during an operation performed via RATs, the management of complications requires careful consideration. A series of *in situ* simulations have been developed around recognizing possible complications and the team process of de-docking the robot allowing for direct surgical intervention (86, 87). These simulations were conducted using all theatre staff as part of the learning environment. This is one of the only true thoracic surgery MDT simulations described in the literature.

## Looking to the future

It is evident the use of simulation is becoming progressively more common throughout thoracic surgery. As highlighted above, developments of technical and non-technical simulations have been reported in the literature in a range of fidelities and modalities though these focus on intraoperative occurrences. The use of virtual reality is also becoming more popular, as is the use of simulations based around new technologies such as RATs.

The aim of this mini-review was to highlight several specific key points regarding the current state of care in thoracic surgery. To summaries:

• Morbidity and mortality in thoracic surgery is not common; however, its management is specific and requires timely action
• The post-operative phase is the period whereby this morbidity and mortality is most likely to occur
• Non-technical failure is the most common cause of post operative morbidity and mortality
• Simulation learning is an emerging and increasingly validated tool.

When reviewing all the above points, one can conclude that targeted simulation training in the management of post operative complications with a focus on non-technical skills can improve patient outcomes. The use of this type of simulation will enable staff to repeatedly, and robustly, practice managing key complications in a controlled learning environment.

In view of the above evidence, it is concerning, that there are no reported studies of ward based post-operative simulations being carried out. Though simulation learning has its flaws, especially in terms of resource use, risk of overconfidence and questions as to translation into clinical practice, the current evidence does suggest an overall positive benefit.

Furthermore, health services throughout the world are developing increasing roles for allied health professionals (AHP). The use of training incorporating these AHPs needs to increase accordingly as they are progressively more relied upon to provide post-operative care.

The literature is unclear as to why simulation training only focuses on intra-operative occurrences. It is likely as these are the most difficult to teach during the real occurrence and also conceivably the area in which the most external scrutiny occurs.

A proposed solution to this is for departments to conduct incident report reviews looking at their range of post-operative complications, their frequencies, and implications to patient outcomes. Subsequently, an index based on frequency, severity and staff confidence in managing these complications could highlight the areas which need the most training. A continuous program of simulations could be created around these occurrences, with specific debrief focusing on common technical and non-technical failures highlighted in the department incident reports. The simulations should be targeted across the breath of staff groups involved in these complications with consistent mentorship over a prolonged period. These simulations should be regularly reviewed to ensure they reflect the current state of complications in said department and ensure they are meeting the training needs of their staff.

As thoracic surgery advances into the next phase of its history, with national screening programs and the potential burdens of disease from chest trauma, vaping, COVD-19 and metastatic disease, a new, dynamic, and highly trained workforce is required. Simulation will inevitably play a role in said workforces' development. Knowing this, time and resources should be placed in creating high-quality, repeatable, and robust MDT simulations to maximize peri-operative care and minimize patient harm.
